# Crying With Laughter: Adapting the Tickling Protocol to Address Individual Differences Among Rats in Their Response to Playful Handling

**DOI:** 10.3389/fvets.2021.677872

**Published:** 2021-06-24

**Authors:** Vincent Bombail, Sarah M. Brown, Tayla J. Hammond, Simone L. Meddle, Birte L. Nielsen, Emma K. L. Tivey, Alistair B. Lawrence

**Affiliations:** ^1^Physiologie de la Nutrition et du Comportement Alimentaire (PNCA), INRAE, Université Paris-Saclay, Paris, France; ^2^The Roslin Institute, The Royal (Dick) School of Veterinary Studies, The University of Edinburgh, Edinburgh, United Kingdom; ^3^Animal and Veterinary Sciences, Scotland's Rural College (SRUC), Edinburgh, United Kingdom; ^4^Modélisation Systémique Appliquée aux Ruminants (MoSAR), INRAE, Université Paris-Saclay, Paris, France; ^5^Universities Federation for Animal Welfare (UFAW), Wheathampstead, United Kingdom

**Keywords:** pinning, playful handling, rough-and-tumble play, social play, rats, tickling, USV

## Introduction

It has been over 20 years since the first scientific papers on rat tickling were published (1, 2). Rats were found to emit ultrasonic vocalizations in the 50-kHz range (hereafter referred to as USVs) when a human performed rapid manual stimulation on their dorsoventral region. Such tickling of rats by a human hand is trying to imitate the rough-and-tumble play seen in young rats of both sexes (3, 4). Emission of USVs by the rat indicates that it enjoys being tickled (5–7), as USVs have been linked with positive emotions (8, 9), are emitted in anticipation of, and during, social play (10, 11), and have been suggested to be homologous to human laughter (12).

**Figure 1:**
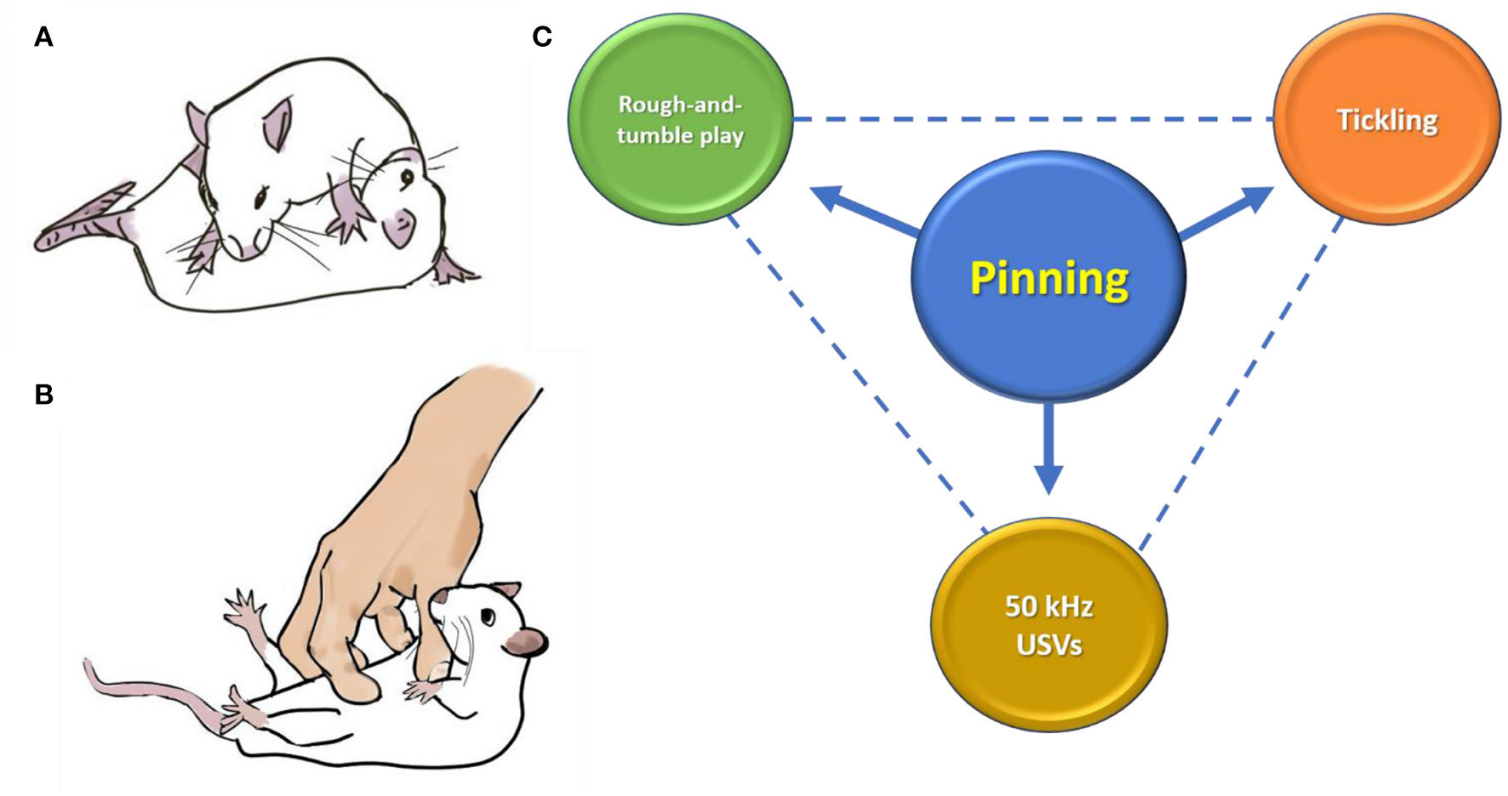
**(A)** Pinning is a component of social play in rats, where one rat has its dorsal surface pinned to the ground, while the other rat is in a dominant posture above. **(B,C)** Pinning has become the main component of the methodology used when tickling rats, as it elicits 50-kHz ultrasonic vocalizations (USVs). Pinning has been used in the past as a means to quantify rough-and-tumble play between rats (14). However, rough-and-tumble play is much more than just pinning, and USVs during pinning are frequent when being tickled by a human (1), but less so when being pinned during play (10, 15). Drawings are by Tayla J. Hammond.

To date, more than 70 scientific articles on rat tickling have been published^1^, and the consensus is that tickling induces positive emotions in rats. Indeed, in a systematic review, LaFollette et al. (13) found that tickling increased USVs and human hand approach behavior, and decreased measures of anxiety in rats. In this Opinion paper, we consider whether current methods of tickling overemphasize the use of pinning (Figure 1) to which there may be a wider response variation than commonly acknowledged. We do not dispute that tickling can be a positive way to handle juvenile rats, but tickling may not always be perceived as a positive interaction by the rat, and we raise the possibility that tickling methods need to be revised. In particular, we suggest incorporating more aspects of play during tickling (increased diversity) and adapting the method to individual rats' responses (increased flexibility) to achieve positive emotions and increased welfare across a wider cohort of rats.

## From Play to Tickling

Play is at the origin of tickling, and the inspiration for Jaak Panksepp to start tickling rats came from his study two decades earlier of the ontogeny of play in this species (14). Play behavior in any species is often complex and unpredictable by nature, making quantification challenging (16–19). By observing adolescent rats engaged in non-aggressive social interactions, Panksepp (14) found that the rats frequently ended up with one rat having their dorsal surface to the ground, with the other rat above holding down (pinning) the supine rat for 2–3 s. Based on these observations, he^1^ suggested that pinning was an easy and objective way to quantify social play in rats.

A comparison of different tickling methods was later published by Panksepp and Burgdorf (1), and the method that induced the most USVs in the 50-kHz range was a full-body tickling with repeated pinning. This was described as “*vigorous whole-body playful tickling (focusing on the ribs and ventral surface), with animals being repeatedly pinned four to six times, throughout the fifteen-second interval*” [p. 361, (1)]. This tickling paradigm (the Panksepp method) has been used widely and is currently employed by a number of different research groups [e.g., (20, 21)].

Panksepp and Burgdorf (1) used the expressions “playful tickling” and “heterospecific handplay” when referring to their rapid manual stimulation of rats by a human hand. This has also been called “heterospecific play” [e.g., (22)] and “playful handling” [e.g., (23)] and is generally referred to simply as “tickling” [e.g., (24)]. The frequent inclusion of the word “play” reflects the original purpose of tickling: to mimic the behavior displayed by rats during play. However, the Panksepp method involves the rat spending most of each 15-s tickling bout on its back, being pinned by the human hand while receiving vigorous finger movements on the ventral area. This contrasts with rat play, which is much more than just pinning, and includes scampering, chasing, sparring, pouncing, darting, dorsal grooming, and wrestling (10, 14, 25, 26).

We suggest that quantifying social play in rats by the occurrence of pinning has given rise to a rat tickling technique (the Panksepp method) that overemphasizes the use of pinning compared with rough-and-tumble play in rats.

## The Role of Pinning

There are two main issues connected with the overuse of pinning during rat tickling. One is that pinning does not occur as frequently during social play as in the tickling method described above. During rough-and-tumble play, group housed rats perform fewer than 10 pins per pair during 5 min of play (14). The other more important issue is that pinning is also the pre-dominant form of rat-to-rat contact during serious fights between adult male rats (27) as frequency and duration of pinning is linked to dominance (14, 28–30), and role reversals are only seen in roughly 30% of play fights among juvenile rats across a variety of strains (31). Pinning during social play in rats has also been referred to as the consummatory phase of play (14).

The occurrence of pinning cannot be used to distinguish play fighting from serious fighting (32), and a supine position cannot be assumed to be submissive or aversive in the context of juvenile play. Play is a means for cooperation as well as competition (33), and most pins during rough-and-tumble play are the result of a full rotation defense tactic of the receiving (pinned) rat in response to a nape attack from the play partner (34). Thus, the adoption of a supine posture during social play is a choice; it peaks during juvenile play as the preferred defensive strategy and declines as rats mature, and rough-and-tumble play takes on a more serious nature (35–37). When pinning is used extensively during tickling, the supine position is not chosen by the rat, and pinning by a human hand is forced rather than offered. During social play, rats have been found to emit more USVs when pinning than when being pinned (15), suggesting that the pinning rat gets greater enjoyment than the pinned rat.

The experience of being pinned by another rat depends on the context of the social interaction (play or fight) and may vary with age. The supine position of the pinned rat during play is a choice, whereas for tickling, the posture of the rat during pinning is involuntary.

## Individual Variability in Ultrasonic Vocalization During Tickling

Ticklishness varies in humans, but people who describe themselves as being extremely ticklish usually do not like it. Two types of tickling exist: *knismesis*, which is when a light touch or stroke evokes a shiver or a twitch, and *gargalesis*, which is a hard, rhythmic probing leading to an intense, often pleasurable sensation (38). Although gargalesis will almost always provoke laughter in humans, this is not necessarily an expression of pleasure, and many people find vigorous tickling aversive (39). Both types of tickling have been tested on rats, and when dorsal knismesis has been compared with gargalesis performed during pinning, the former elicits fewer USVs (6, 40). However, the type of dorsal knismesis used in these studies, i.e., repeated gentle touching of the rat's dorsal surface, is not comparable with the types of touch and movements experienced by rats during play.

Rats show large individual variability in their expression of 50-kHz USVs (21, 41, 42), with some rats not emitting any when tickled (1, 43). Using an affective bias test, Hinchcliffe et al. (21) demonstrated that USVs are correlated with the level of positive affect on a gradual scale, so that rats producing frequent USVs during tickling can be assumed to like it more than their less vocal conspecifics. High callers show positive cognitive bias when tested after tickling (44). Rats can be genetically selected based on their USV frequency during tickling (2, 41, 45), and rats selected for low USV emission display more pins during social play (46). This is robust evidence that tickling to induce positive emotions needs to take into account the individual response in the level of rat enjoyment from being tickled.

The enjoyment of being tickled varies among rats. The use of pinning may not always contribute positively to the tickling experience for all rats, and a tickling protocol with extensive use of pinning does not allow the rat to express the degree to which it finds the handling enjoyable or even aversive.

## How Can We Simulate Play Better During Tickling?

The Panksepp method [systematized by (20) and (22)] is widely used and consists of structured and repeated pinning, which makes it difficult both to assess the affective state of the rat and adapt the tickling to its behavior. We would argue that the resemblance to rough-and-tumble play is lost when the method becomes predictable, follows a protocol, and does not take into account individual variation in the response of the rats. If pinning is aversive to some rats, the inclusion of more aspects of rat play when tickling would have a higher likelihood of being pleasurable for more rats. Schwarting et al. (43) was among the first to use different types of tactile manipulations to tickle rats. However, this study used adult rats, with tickling sessions lasting 10 min, and without adjusting the manipulations to the rat's response. In contrast, Bombail et al. (5) used various tactile manipulations resembling the elements of play listed in the section on *From play to tickling*, and with their type and vigor adjusted to the response of each rat, and found USVs increasing from 83 to 233 per minute, on average, from the first to the fifth playful handling session. In comparison, using the Panksepp method, Burgdorf et al. (45) recorded around 90 USVs/min during the fourth tickling session in their randomly selected line. This suggests that by making tickling more diverse and more flexible instead of focusing on pinning, we can cater for the likes (and avoid the dislikes) of more rats.

USVs during rough-and-tumble play in rats differ between different aspects of play, e.g., Kisko et al. (10) found significantly more USVs emitted during wrestling and chasing than during pinning. It is also yet unknown if USVs can be “forced” when using pinning during tickling in the same way as when humans cannot prevent laughing when being tickled forcefully, even if they do not like it. Several studies indicate that these vocalizations serve a function for the nature of play. Rats that cannot vocalize play less and with role reversals more than halved (47), and wrestling is reduced in play fighting between deaf rats (48). Burke et al. (49) found that some calls were associated with a particular play behavior. Certain calls affect the likelihood of different aspects of play occurring, indicating that some calls communicate specific information to the play partner (50). However, most studies of tickling do not report the type of vocalizations, only total USV counts. The USVs emitted during tickling are not in the range of human hearing, so without special equipment, the evaluation of the rat's enjoyment is based purely on the rat's behavioral response.

We suggest that more components observed during social play in rats are included during playful handling while reducing the use of pinning. This would be a more inclusive tickling method that is more playlike and likely to be a pleasant experience for more rats, including individuals that do not enjoy being pinned.

## Conclusions and Perspectives

There is a clear evidence that the majority of rats enjoy playful handling by a human. The findings that (1) pinning frequency can be used to quantify play, (2) 50-kHz USVs are associated with enjoyment, and (3) using pinning when tickling provokes USVs may have led to tickling protocols focused on the use of pinning and the assumption that these are always enjoyable. We question this notion.

We suggest that the expression “*playful handling*” should be used when the interaction between the rat and the human hand resembles the full repertoire of rough-and-tumble play between two rats, i.e., manipulations involving some or all of dorsal and ventral tickling; chasing, sparring, and wrestling by both hand and rat, and which may include some pinning. It should be flexible and aim to be somewhat unpredictable (38), as the human participant gauge what the rat would find the most enjoyable. “*Tickling*” could then be used to describe the Panksepp method (20, 22), with repeated pinning and vigorous finger movements on the ventral area of the rat. This method would be a standardized treatment of a group of rats, similar to giving the same dosage of a drug to all subjects. However, such tickling does not have the same effect on all animals, and appropriate controls would therefore be difficult to generate.

Our description of playful handling may come across as subjective, but playful handling can be used scientifically, as demonstrated in a number of trials (5, 51, 52). Just like we adjust drug dosage to body weight, or train rats for different durations depending on their learning skills, maximizing enjoyment of playful handling requires adaptation to the individual rat's behavioral responses to interactions with the human hand. More diversity and flexibility in the tickle paradigm is called for, allowing the experimenter to respond sensitively to the rat's behavior with the aim to achieve equitable affective experiences for all rats. Taking individual variation into account is increasingly being promoted in the scientific literature, and by being more inclusive of individual differences, playful handling can improve the welfare of all rats in our care by actively promoting positive affective states (53–55).

## Author Contributions

VB, BN, and AL initiated the discussion of the subject and the writing of this paper. SB, TH, SM, and ET contributed significantly to the discussion of the subject, and the development, writing, and final version of this paper. All authors contributed to the article and approved the submitted version.

## Conflict of Interest

The authors declare that the research was conducted in the absence of any commercial or financial relationships that could be construed as a potential conflict of interest.

## Footnotes

^1^Web of Science search with [(rat OR rats) AND tickl^*^] in title, abstract, or keywords omitting meeting abstracts.
